# Development and Comparison of Two 3D-Printed Scaffolds of Biosilica from Marine Sponges for Bone Tissue Engineering

**DOI:** 10.1021/acsomega.4c11383

**Published:** 2025-05-15

**Authors:** Giovanna do Espirito Santo, Amanda de Souza, Gustavo Oliva Amaral, Amanda Sardeli Alqualo, Karolyne dos Santos Jorge Sousa, Beatriz Louise Mendes Viegas, João Paulo dos Santos Prado, Francisco Vieira dos Santos, Daniel Souza Correa, Renata Neves Granito, Ana Claudia Muniz Rennó

**Affiliations:** † Department of Biosciences, 28105 Federal University of São Paulo (UNIFESP), Silva Jardim Street, 136, Santos, SP 11015020, Brazil; ‡ 564899 Nanotechnology National Laboratory for Agriculture (LNNA), EMBRAPA Instrumentação, São Carlos, SP 13560-970, Brazil; § Materials Engineering Department, Sao Carlos School of Engineering (EESC), 28133 University of Sao Paulo (USP), Sao Carlos, SP 13563−120, Brazil

## Abstract

This study compared the physicochemical characteristics and biological effects of two 3D-printed biosilica (BS) scaffolds (grid and gyroid). The methods included scanning electron microscopy (SEM), porosity, mass loss, pH assessment, Fourier transform infrared spectroscopy (FTIR), and energy dispersive X-ray spectroscopy (EDS). The mechanical evaluation involved a compression test, and the in vitro tests used cell adhesion assays with osteoblastic and fibroblastic cell lines. SEM showed BS spicules in both models at day 0 with signs of degradation throughout the experimental immersion periods, forming a homogeneous network with interaction with alginate. Porosity measurements showed an average of 85.9% ± 0.9 for the grid model and 83.6% ± 0.7 for the gyroid model. The gyroid model demonstrated higher values in the compression test, a decrease in pH on day 1, and no difference for both models on days 3, 7, and 14. Mass loss was greatest in the gyroid model on day 21. FTIR tests showed characteristic peaks for ALG and BS. EDS detected silica (Si), chlorine (Cl), and calcium (Ca). In the cell adhesion assay, both models supported the adhesion and proliferation of L929 (fibroblast) and MC3T3-E1 (osteoblastic) cells, with the gyroid model showing better elongation and cell morphology. Overall, the gyroid model showed better physicochemical properties, higher mechanical strength, and improved biological performance compared to the grid model, making it a promising option for tissue engineering.

## Graphical abstract

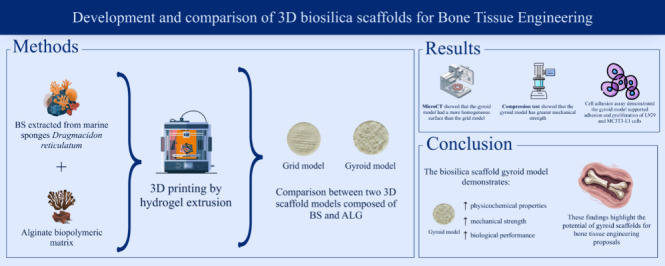

## Introduction

The incidence of fractures has significantly increased in the last years and represents a public health issue around the world and a serious economic burden.^1^ In general, most of the fractures heal by themselves, but in specific situations, such as in fractures related to osteoporosis or traumatic fractures with great extension, the process of healing may be impaired, leading to a delay in the process of consolidation or even in the occurrence of nonconsolidated fractures.^2^ In this context, surgical procedures are the treatment of choice, having the aim of fixing the fractures and/or implanting biomaterial bone grafts for bone healing.^3,4^

Biomaterial bone grafts have been considered one of the effective therapeutic interventions for bone tissue replacement due to their potential for stimulating tissue growth and bone consolidation.^5,6^ To date, a series of different classes of biomaterials for bone regeneration have been investigated, including synthetic and natural biomaterials.^7^ From the class of natural biomaterials, biocompounds extracted from marine sponges have been demonstrating a remarkable potential to be used in tissue engineering.^8–11^ One of the main components of marine sponges is biosilica (BS) (glassy amorphous silica- SiO_2_), which has emerged as a promising raw material for bone grafts.^12^ BS is part of the inorganic skeleton of marine sponges, and it is formed by an enzymatic and silicatein-mediated reaction.^13^ Some authors have extracted BS from sponges and demonstrated, through in vitro studies, evidence of the osteogenic potential of BS and its ability to stimulate mineralization, to upregulate the expression of genes related to bone cell differentiation, and to increase cell proliferation.^14^ Gabbai-Armelin et al.^14^ have demonstrated in an in vitro study that BS had a positive influence on MC3T3-E1 cell viability and was able to increase Runx2 and BMP4 gene expression, indicating a potential use of BS to be used for tissue engineering applications.

Moreover, the structure and morphology of the bone graft scaffold are crucial factors that need to be carefully considered to effectively promote healing. To achieve the success of the bone implant, bone grafts need to present an adequate porosity with interconnected pores and high mechanical properties.^15^ In this context, additive manufacturing, also known as 3D printing, is an innovative technology that has been widely used for the rapid prototyping scaffolds.^16,17^ 3D-printed scaffolds offer many advantages over other manufacturing techniques, allowing the fabrication of patient-specific bone grafts, with controlled porosity, shape, and size, resulting in unique structures capable of promoting bone ingrowth.^18^ Using this innovative printing technique, many different structures for manufacturing scaffolds can be obtained, being one of the most common the grid model.^18,19^ They are composed of superimposed multiple layers, providing an appropriate environment capable of supporting cell growth and proliferation.^19^ Another model of 3D-printed scaffolds widely used is the gyroid model, which is composed of a structure with parallel and perpendicular wavy filaments and a well-distributed arrangement of pores.^20^ Qi et al.^21^ demonstrated that 3D-printed gyroid scaffolds manufactured with β-TCP and magnesium oxide promoted the osteogenic differentiation of bone marrow mesenchymal stem cells (BMSCs) and angiogenic differentiation of endothelial progenitor cells (EPCs).

Despite all the advantages of the use of the 3D printing technique for scaffold manufacturing, such as the print accuracy and rapid fabrication of porous structures, another important factor that is essential for reaching an optimized effect for stimulating tissue metabolism is the choice of the right biomaterial for printing ink production.^22^ As the need for the development of more efficient bone grafts for improving the process of bone ingrowth and healing, the fabrication of 3D-printed scaffolds with a highly active natural ceramic (such as BS), in two models, is an excellent approach trying to accomplish all the different requirements for reaching the success of bone remodeling. It is worthwhile to emphasize that the scaffolds studied in the present work were manufactured with the association of BS and alginate (ALG), which is also a biocompatible and biodegradable material, mimicking the extracellular matrix.^23–25^ The combination of BS and ALG may offer a synergistic effect, with BS being able to stimulate bone ingrowth while ALG provides a suitable structure for cell adhesion and tissue integration. In this context, 3D-printed scaffolds based on BS/ALG constitute an innovative approach for bone tissue engineering proposals.

Therefore, the comparative study of both models having BS as the base material can contribute to the understanding of which format is more appropriate for the construction of scaffolds for stimulating bone repair. The hypothesis is that the grid and gyroid 3D-printed scaffolds made of marine sponge BS and ALG would exhibit properties arising from their composition and geometries, capable of stimulating cell proliferation and adhesion differently. In this context, this study aimed to fabricate two different 3D-printed scaffold models made with ALG and marine sponge BS, grid, and gyroid, and to study their physicochemical and mechanical characteristics and their biological effects through in vitro tests.

## Materials and Methods

### Materials

Sodium hypochlorite (NaClO, 15% v/v, CAS: 7681-52-9), nitric acid (HNO_3_, 65%, v/v, CAS: 7697-37-2), sulfuric acid (H_2_SO_4_, 98% v/v, CAS: 7664-93-9), sodium alginate (ALG, CAS: 9005-38-3), and calcium chloride (CaCl_2_ ≥ 99% P.A, CAS: 10043-52-4) were all supplied by Sigma-Aldrich (San Luis, Missouri, EUA). Minimum essential medium alpha (MEM-α), Fetal bovine serum (FBS), and Dulbecco’s modified Eagle’s medium (DMEM) were sourced from Vitrocell Embriolife (Campinas, São Paulo, Brazil). Phalloidin Alexa Fluor 488 was provided by Life Technologies (Oregon, USA). Perfluoroalkoxyalkanes (PFA) came from Synth (Diadema, São Paulo, Brazil), and DAPI was supplied by Thermo Fisher Scientific (Waltham, Massachusetts, EUA).

### BS Extraction

BS was extracted from the marine sponge species *Dragmacidon reticulatum* collected on the site of Praia Grande, São Sebastião, São Paulo, Brazil. The samples were washed with distilled water to remove any unwanted material from the primary collection and then, with a scalpel blade, cut into pieces of approximately 1 × 1 cm^2^ and immersed in 5% (v/v) NaClO until the degradation of the organic material.^26^ After this stage, samples were washed with distilled water to remove NaClO and HNO_3_, and H_2_SO_4_ (1:4) was added to dissolve the residual organic part. After 24 h, the BS particles were decanted, and distilled water was added until it reached a pH > 6 (Tecnal, TEC-51, Piracicaba, São Paulo, Brazil). Finally, the obtained BS powder was dried in an oven (SPLabor, Presidente Prudente, São Paulo, Brazil) at 37 °C and sieved (A Bronzinox, Santo Amaro, São Paulo, Brazil) to produce particles around 106 μm in size. BS powder was then stored in a Falcon tube and kept under a vacuum.

### Printing Ink Protocol

The ink ratio for the 3D printing was set at 70:30, with 70% BS and 30% ALG. For this proposal, 9.333 g of BS and 4 g of ALG were weighed with an analytical balance (Bel Engineering, M314Ai model, Bel Engineering S.r.l., Monza, Itália). BS was weighed and homogenized with 50 mL of distilled water in a Falcon tube in a vortex (Gehaka, AV-1, Real Parque São Paulo, São Paulo, Brazil) to avoid future clogging of the printing needle due to the BS particles and stirring for 1 min. The mixture was transferred to a beaker, covered with parafilm to prevent evaporation of the solution, placed under a magnetic stirrer (Tecnal, TE-0851, Piracicaba, São Paulo, Brazil), and heated with an additional 50 mL of distilled water until reaching 65–75 °C, at a speed of 120 rpm. When the temperature was reached, ALG was added slowly and then homogenized under the same temperature range for 1 h. After this period, a homogeneous hydrogel was formed, and the printing ink was subjected to 25 mL of a primary cross-linker with 1% (w/v) CaCl_2_, stirred, and finally stored under refrigeration.

### 3D Printing Protocol for BS Scaffolds

Scaffolds were printed by the 3D printing technique performed by extrusion, since through this approach, it is possible to obtain thin three-dimensional structures, with controlled porosity and detailed control over the final shape of the construct. This method is based on the use of a biocompatible hydrogel, which is extruded through a printing nozzle in a predefined pattern, to be deposited layer by layer and form the appropriate structure.^27,28^ Furthermore, during 3D printing by extrusion, flexibility in material selection can be achieved by the possible use of hydrogels that have desired characteristics, such as a controlled degradation rate and the ability to host bioactive agents.^29^

For the manufacture of the 3D-printed scaffold, a computational model was developed using the TinkerCAD web application (Autodesk, Inc., San Rafael, California, United States), specifying a scaffold with a 20 mm diameter and a 3 mm thickness (Figure 1A,B).

**Figure 1:**
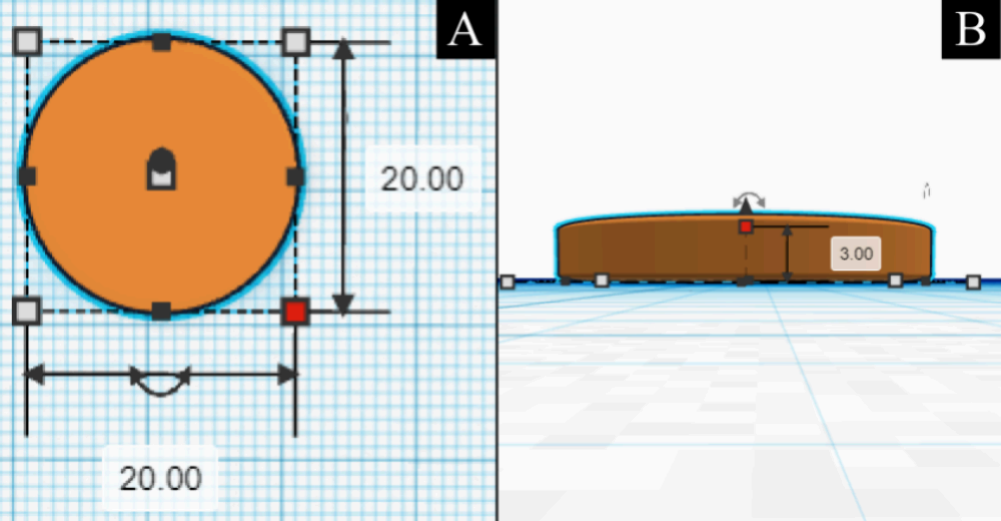
Defining the diameter and thickness of scaffolds in the TinkerCAD web application. (A) Scaffold diameter and (B) scaffold height.

The model was then imported into Cura Ultimaker 5.8 software (Ultimaker, Utrecht, The Netherlands) for slicing. At this stage the printing parameters were defined as gyroid (Figure 2A) and gyroid (Figure 2B) fillings, the height of each layer at 0.75 mm (totaling 4 in both models (Figure 2C)), distance between filaments of 1.5 mm, printing speed of 10 mm/s, printing flow at 10%.

**Figure 2:**
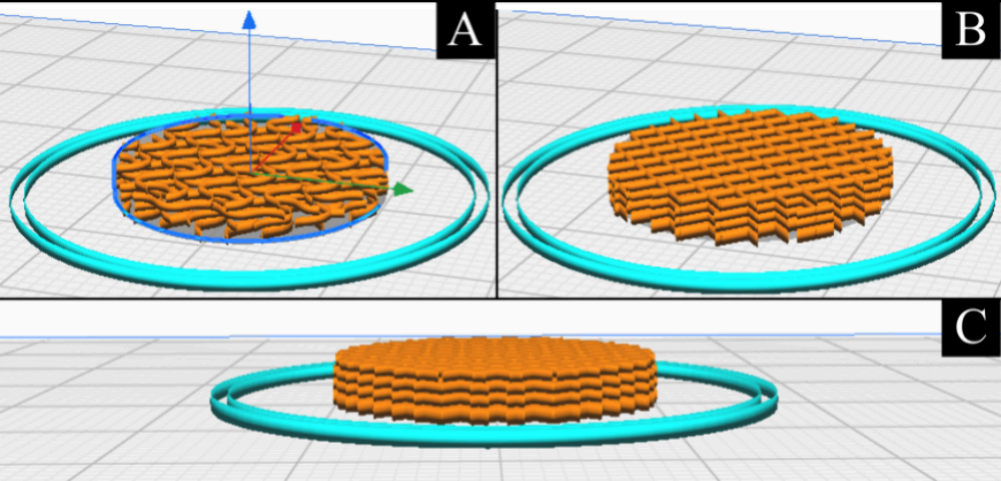
Scaffold filling models: (A) Grid model, (B) gyroid model, and (C) demonstration of the four layers.

Then, the ink was loaded into a 5 mL syringe and inserted into the extruder of the 3D printer (Educational Starter, 3D Biotechnologies Solutions, Campinas, Brazil), with the scaffolds being printed layer by layer with a 0.9 mm diameter needle.

Subsequently, the printed structures underwent a secondary cross-linking process using 25 mL of 2% (w/v) CaCl_2_ and were then immersed for 25 min. They were then briefly rinsed with distilled water and frozen for subsequent partial freeze-drying (Terroni Equipamentos Cientifiques Ltd.a., São Carlos, São Paulo, Brazil) for 1 h, then dried in an oven (SPLabor, SP-100/150 model, Presidente Prudente, São Paulo, Brazil), in order not to weaken the three-dimensional structure of the scaffolds, and finally subjected to tertiary cross-linking under UV light (403 nm) (Educational Starter, 3D Biotechnologies Solutions, Campinas, Brazil) for 10 min on each side of the scaffolds.

### Scanning Electron Microscopy (SEM)

Scanning electron microscopy (SEM, model JSM-6610LV, JEOL Ltd., Akishima, Tokyo, Japan) was used for analyzing the morphology of the produced scaffolds. The scaffolds were evaluated without incubation and after 1 and 21 days of incubation in phosphate-buffered saline (PBS). For the SEM analysis, the samples were placed on conductive carbon tape and covered with a thin gold layer (20 nm) by using a sputter coater (Balzers, model SDS 050, Oerlikon, Balzers, Liechtenstein). The analysis was conducted by using a secondary electron (SE) detector, and an accelerating voltage of 10 kV was applied to ensure sufficient resolution for surface morphology visualization.

### Porosity

The methodology used was based on the Archimedes Principle, as previously described in Camilo et al.^30^ For apparent porosity, 5 mL of distilled water was added to a 10 mL graduated cylinder and weighed (*m*_1_) by using an analytical balance. Subsequently, the scaffold was placed in another graduated cylinder and reweighed (*m*_2_). Once the balance stabilized, the value was recorded (*m*_3_). The analysis was performed with six scaffolds of each model and with the equation derived as follows:

$$\%\mathrm{Porosity}=\frac{\frac{m_{1}-m_{3}}{\det}}{\left[\left(\frac{m_{1}}{\det}\right)+\left(\frac{m_{\mathrm{scf}}}{\mathrm{dscf}}\right)\right]-\left(\frac{m_{3}}{\det}\right)}\times 100$$

### Compression Test

To evaluate the mechanical properties of the scaffolds, the maximum tensile stress was measured using a Universal Testing Machine (Model 5582, Instron, Norwood, Massachusetts, USA) with ASTM D3967 standards, with a travel speed of 0.5 mm/min. The analysis was performed in triplicate, and the results were obtained using eq [Disp-formula ueq2]:

1 $$\sigma =\frac{2F_{\max}}{\pi Dt}$$

The stress applied to the material is represented by σ, *F*_max_ is the highest force that the material can bear before breaking, *D* is the diameter, and *t* is the thickness of the specimen.

### Mass Loss and pH Assessment

For the mass loss test, the produced scaffolds were individually weighed to determine the initial mass before being divided into Falcon tubes, according to the experimental time periods of 1, 3, 7, and 14 days (*n* = 5 per group and per experimental period). They were then immersed in PBS (10 mM, pH 7.4) and incubated in a 37 °C oven. After each experimental period, scaffolds were removed, oven-dried at 37 °C for 24 h, and weighed to establish their final mass. The leftover PBS was measured with a pH meter using the same technique.

### Fourier-Transform Infrared Spectroscopy (FTIR)

To elucidate the chemical bonds present in the scaffolds produced, the FTIR technique (Thermo Nicolet Nexus 4000, Thermo Fisher Scientific Inc., Waltham, MA, USA) was conducted. The spectra were acquired in the range of 400–4000 cm^–1^ with a resolution of 2 cm^–1^.

### Energy-Dispersive X-ray Spectroscopy (EDS)

The relative quantification of atomic elements present in the scaffolds was determined (SEM-JEOL, model JSM-6610LV, Shimadzu Corp., Tokyo, Japan). The samples were immersed in an SBF solution for 0, 1, 3, 7, 14, and 21 days. They were then removed and dried in an oven at 37 °C until completely dry. For this analysis, the samples passed through an X-ray tube with a Rh anode operating at 5–50 kV and 1–1000 microA.

### In Vitro Studies

The biological response of the BS scaffolds was assessed by culturing osteoblast cells (MC3T3-E1) and murine fibroblast cells (L929), obtained from the Rio de Janeiro Cell Bank (BCRJ), following the ISO standard 10993–5:2009 guidelines. These cell types were cultured in bottles using α-MEM and DMEM supplemented with 10% FBS and 1% antibiotic-antimycotic solution at 37 °C in a humidified atmosphere of 5% CO_2_ for the respective cells. They were maintained at subconfluent densities and passaged weekly until use.

### Cell Adhesion Assay

The MC3T3-E1 (osteoblasts) and L929 (murine fibroblasts) cell lines were seeded (1 × 10^6^ cells/mL) on the surface of the scaffolds per premoistened with the culture medium, followed by an incubation time of 3 h (5% CO_2_, 37 °C, and 95% humidity).

Cell adhesion was observed by confocal microscopy (SP8 AOBS Tandem Scanner, Leica Microsystems, Wetzlar, Germany) at 1, 3, 7, and 14 days after seeding (*n* = 5 photographed fields for each scaffold model according to experimental time). Scaffolds were subjected to a three-step washing process with a PBS solution to remove the cells that were not firmly adhered to the surface of the scaffolds. They were then immersed in a 4% PFA solution for cell fixation of the ones adhered to the surface of the samples. Subsequently, cells were stained with Phalloidin Alexa Fluor 488 to identify the presence of actin filaments and DAPI to analyze the nuclear deoxyribonucleic acid (DNA).

### Statistical Analysis

The distribution of variables was tested using Shapiro-Wilk’s normality test. Parametric variables, when comparing groups, underwent a two-way analysis of variance (ANOVA). For nonparametric variables, the analysis involved Welch’s *t*-test. The statistical software used was GraphPad Prism version 8.0 (GraphPad Software Inc., La Jolla, CA, USA), and a significance level of *p* ≤ 0.05 was adopted, followed by the Bonferroni posthoc test.

## Results and Discussion

### Characterization of Scaffolds

#### SEM Analysis

Figure 3 demonstrates the SEM micrographs of the grid and gyroid scaffolds before and after incubation. Digital images of the grid and gyroid models, obtained before immersion, can be seen in Figure 3A,B, respectively. Differences in surface topography were observed, and the grid model presented a more regular and grooved surface, while the gyroid model exhibited a more wavy and porous texture. Figure 3C,D demonstrate that at day 0 (without incubation), BS spicules can be seen for both grid and gyroid models. Moreover, both samples presented pores distributed throughout the samples. On day 1 after immersion, for both models, the spicules were still visible but partially incorporated into the ALG matrix, while some agglomerations were present (Figure 3E,F). After 21 days of incubation, the integrity of BS spicules was no longer seen for both samples, showing a significant degradation of the material and forming a homogeneous net with the ALG particles (Figure 3G,H).

**Figure 3:**
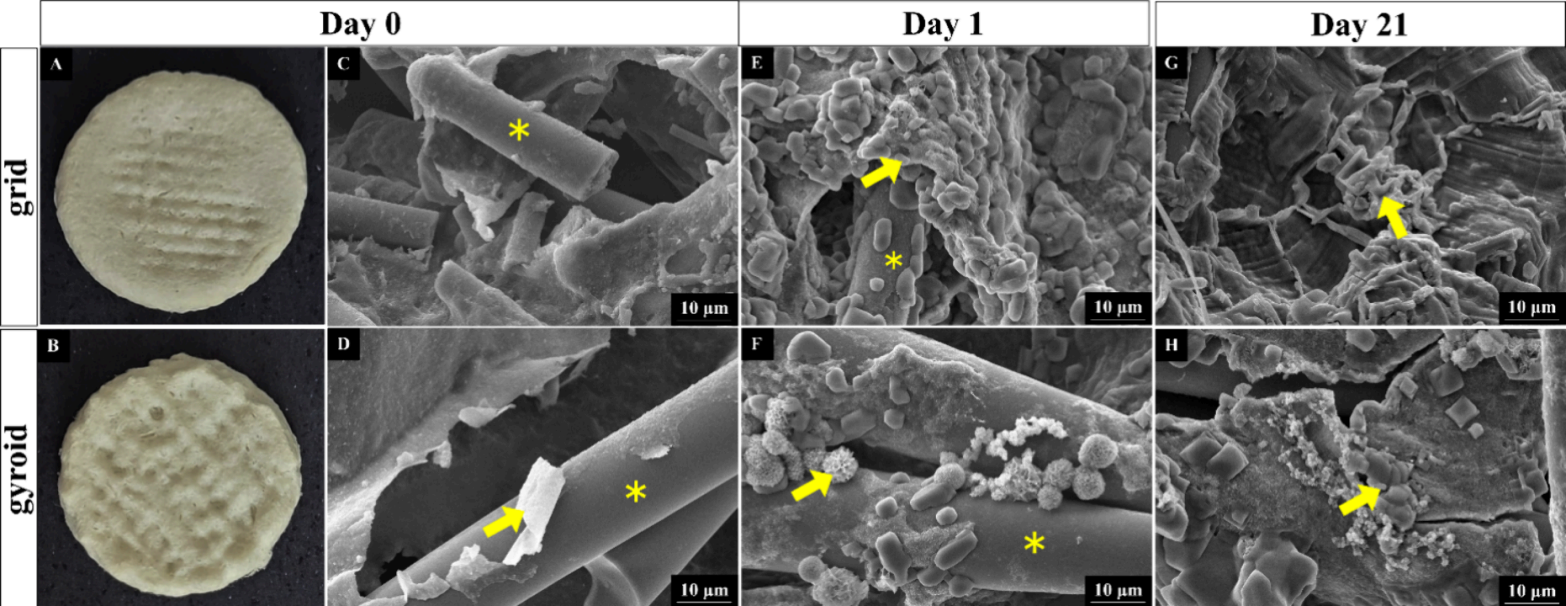
SEM images of the surface for the grid and gyroid models (2500 × magnification) and digital images of the grid and gyroid models (A, B); (C, D) images representing the models without incubation in PBS solution; (E, F) after 1 day incubated in PBS solution; (G, H) after 21 days incubated in PBS solution. The scale bar represents 10 μm (* indicates BS spicules, and → yellow indicates agglomerations incorporated into the ALG network).

#### Porosity

The average porosity values can be seen in Figure 4. For the grid model, the mean value was 85.9 ± 0.9% and for the gyroid model was 83.6 ± 0.7%. Also, a significant statistical difference was observed between the mean values for the porosity for both models.

**Figure 4:**
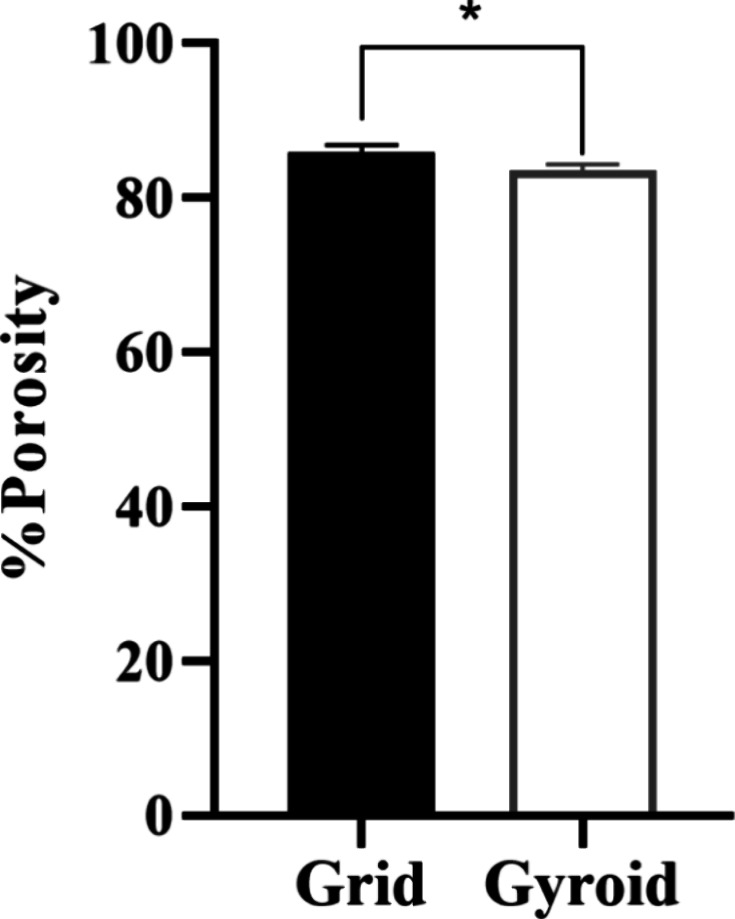
Comparison of the Porosity between the grid and gyroid models. Statistically significant differences are indicated by (*) (Two-way ANOVA, *p* < 0.05).

#### Compression Test

Regarding the compression test, Table 1 presents the average values obtained in the mechanical compression test. Therefore, this analysis showed that the maximum compression capacity values were 4.90 ± 0.36 and 11.71 ± 1.21 N for the grid and gyroid models, respectively, with a statistical difference. In addition, the values equivalent to how much load each of the models can withstand before rupture were also presented, with the gyroid model showing a greater capacity to withstand loads, 431.05 ± 78.28 kPa, when compared to the grid model, which showed lower values, 109.26 ± 74.52 kPa, with a statistical difference between the two models.

**Table 1:** Values of the Mechanical Compression Test for the 3D-Printed Scaffolds^a^

3D printed scaffolds models	*F*_max_ (N)	σ_max_ (kPa)
grid	4.90 ± 0.36	109.26 ± 74.52
gyroid	11.71 ± 1.21 (*)	431.05 ± 78.28 (*)

a Statistically significant differences are indicated by (*) in *F*_max_ and σ_max_ (Two-way ANOVA, *p* < 0.05).

#### pH Evaluation

As shown in Figure 5, both models showed a decrease in pH values during the experimental periods. On day 1, the pH values were 6.0 ± 0.02 for the grid model and 5.7 ± 0.2 for the gyroid model, with a significant difference between both groups. On day 3, the grid model presented a pH of 5.9 ± 0.1 and the gyroid model a value of 5.7 ± 0.1 (with a significant difference). On day 7, the pH of the grid model decreased to 5.5 ± 0.1, and that of the gyroid model decreased to 5.5 ± 0.02. On day 14, these values decreased even further, to 5.4 ± 0.1 and 5.5 ± 0.03, respectively.

**Figure 5:**
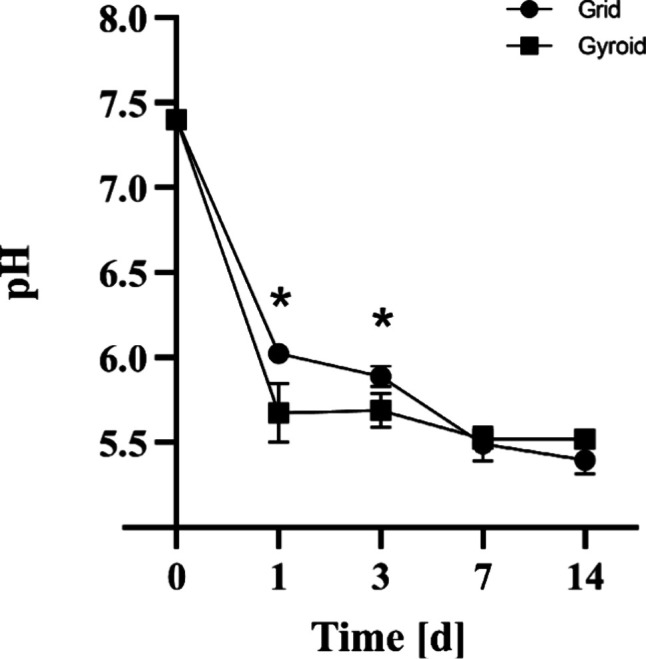
pH Values of grid and gyroid models as a function of time period in PBS. Statistically significant differences (asterisks) were observed on days 1 and 3 (Two-way ANOVA, *p* < 0.05).

#### Mass Loss

Both grid and gyroid models showed progressive degradation during the experimental period (Figure 6). On day 0, the grid model started with an average weight of 0.103 g ± 0.001, while the gyroid model had an average weight of 0.105 g ± 0.016. From this, on day 1, a mass decrease to 99 ± 1% for the grid model and 97 ± 1% for the gyroid model of their initial value. On day 3, the grid model remained more stable, presenting 97 ± 1%, while the gyroid model presented 89 ± 3%. On day 7, the grid model presented 94 ± 1% of the initial mass, while the gyroid model decreased to 87 ± 2%. On day 14, the grid model presented 93 ± 1% of the initial mass, while the gyroid model presented 70 ± 3%. Statistically significant differences were observed between the values found for both models on days 3, 7, and 14.

**Figure 6:**
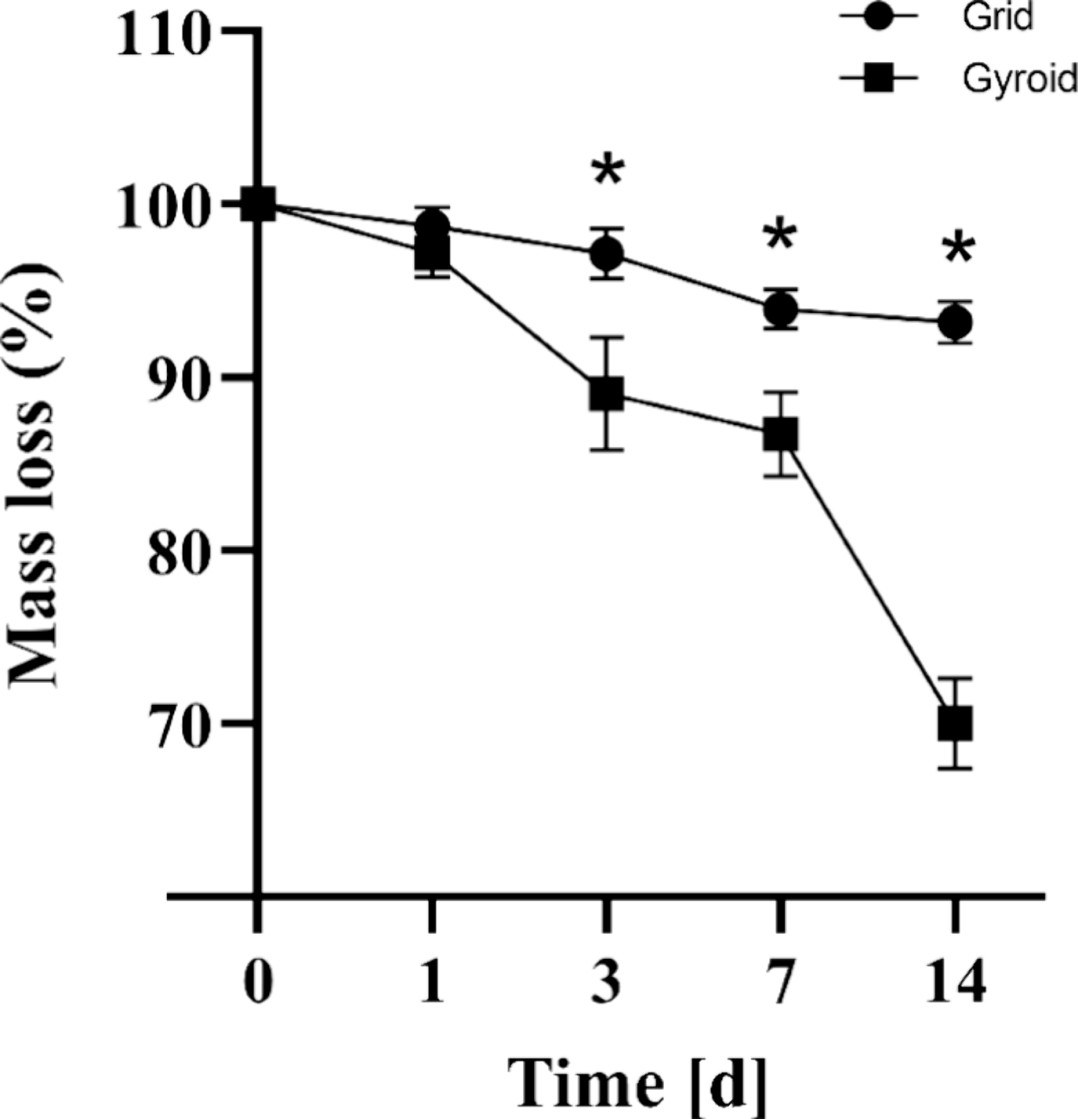
Mass loss, in percentage, for grid and gyroid models as a function of time period in PBS. Statistically significant differences are indicated by (*) on days 3, 7, and 14 (Two-way ANOVA, *p* < 0.05).

#### FTIR Analysis

The chemical bonds present in the chemical composition of the scaffolds after the manufacturing, cross-linking, and drying stages were described and depicted in Figure 7 and Table 2. Four characteristic peaks of ALG present in the scaffolds were observed, including a stretching vibration of O–H at 3421 cm^–1^.^31^ Additionally, vibrations of asymmetric and symmetric stretching of C=O were present at 1628 cm^–1^.^32^ The final two peaks corresponded to C–O–H and C–O–C in the 1404 and 1022 cm^–1^, respectively.^32^ Meanwhile, the peaks evidencing the silicon group corresponded to the stretching vibration of the Si–O–Si group at the 1096 cm^–1^ band.^14^ There was also a bending vibration of the Si–O group at the 789 cm^–1^ band, and finally, an out-of-plane bending vibration corresponding to the 1096 cm^–1^ band.^14,33^

**Figure 7:**
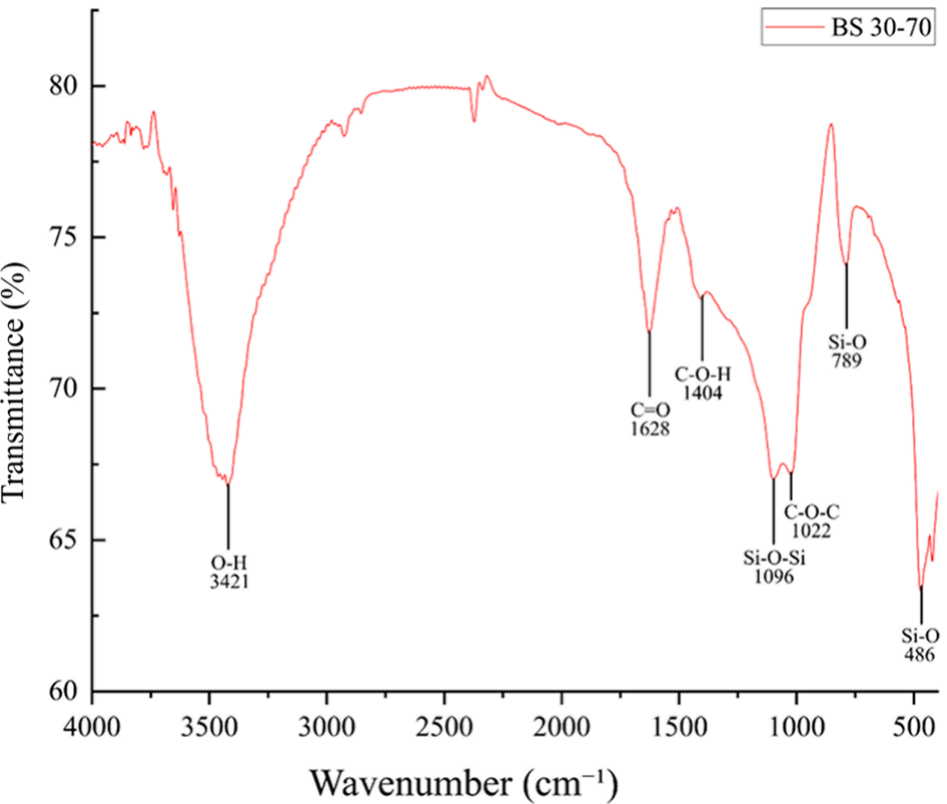
Bands obtained via FTIR indicate the presence of characteristic functional groups of ALG and BS.

**Table 2:** Band was Obtained through FTIR Analysis of Scaffolds

wavenumber (cm^–1^)	functional group	references
3421	O–H stretching vibration	Lach et al.,^31^
1628	C=O asymmetrical and symmetric stretching vibrations	Belattmania et al.,^32^
1404	C–O–H	Belattmania et al.,^32^
1022	C–O–C	Belattmania et al.,^32^
1096	Si–O–Si stretching	Gabbai-Armelin et al.,^14^
789	Si–O bending vibrations	Ellerbrock et al., and Gabbai-Armelin et al.,^14,33^
486	Si–O out-of-plane bending vibrations	Ellerbrock et al.,^33^

#### EDS Analysis

The relative amounts of the elements carbon (C), oxygen (O), silicon (Si), and calcium (Ca) presented in the samples were measured and are presented in Figure 8. It was observed that on day 0 (without incubation in SBF solution), the gyroid model exhibited elements C, O, and Si, similarly to the grid model, which also showed the presence of Cl and Ca. On Day 1, there was a decrease in C and O but an increase in the other elements (Si, Cl, and Ca). The grid model also showed a significant increase in the same elements. Starting from day 14, the gyroid model showed fewer elements than the grid model, especially in the Si element. There were statistical differences between the groups on the following days and elements: on day 0 for the C element; on day 1 for the Cl element; on day 3 for the C and Ca elements; on day 14 for the C, O, Si, and Cl elements; and on day 21 for the C and Cl elements. On day 7, there was no statistical difference.

**Figure 8:**
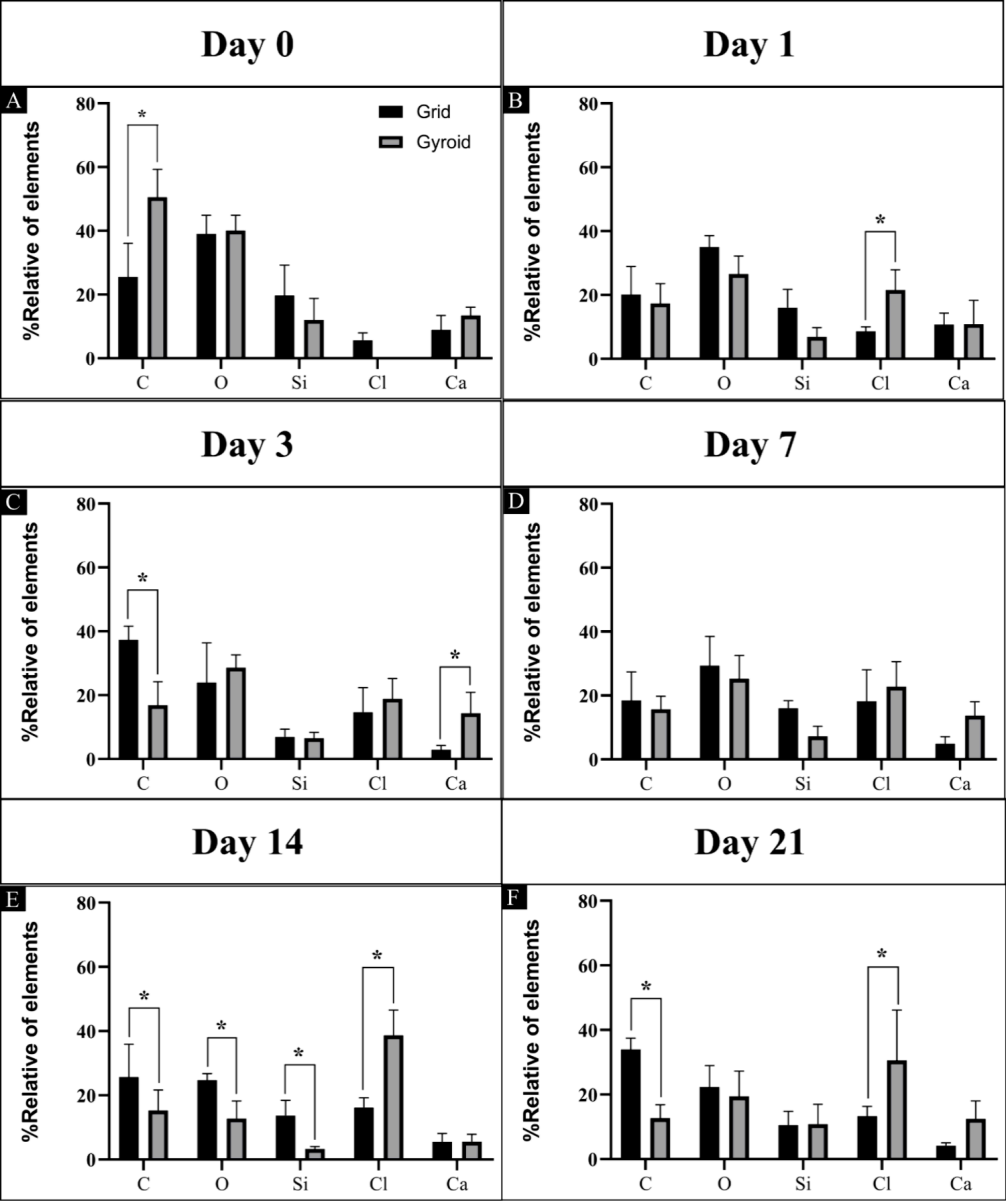
EDS semiquantitative analysis of elements contained in the grid and gyroid models. (A) Quantification on day 0, without incubation in the SBF solution; (B–F) with incubations in the PBS solution at times 1, 3, 7, 14, and 21, respectively. * Statistical difference (Two-way ANOVA, *p* < 0.05).

### In Vitro Studies

#### Cell Adhesion Assay

Figure 9 presents the results obtained by confocal microscopy analysis. Initially, on day 1, both scaffold models displayed reduced cell adhesion, with round-shaped cells appearing more scattered. By day 3, a notable difference could be observed, as the number of cells remarkably increased in both models. Moreover, in the gyroid model, adherent cells started to spread out along the scaffolds. Continuing to day 7, the cell number increased in both models, indicating cell proliferation. In addition, during this experimental period, changes in cell shape and cytoskeletal rearrangements were more evident, with no visible difference concerning cell spreading in grid and gyroid models. However, on day 14, cells returned to their initial round morphology on the grid model scaffolds, while they remained elongated, still displaying the characteristic stretched fibroblastic morphology on the gyroid-shaped scaffolds.

**Figure 9:**
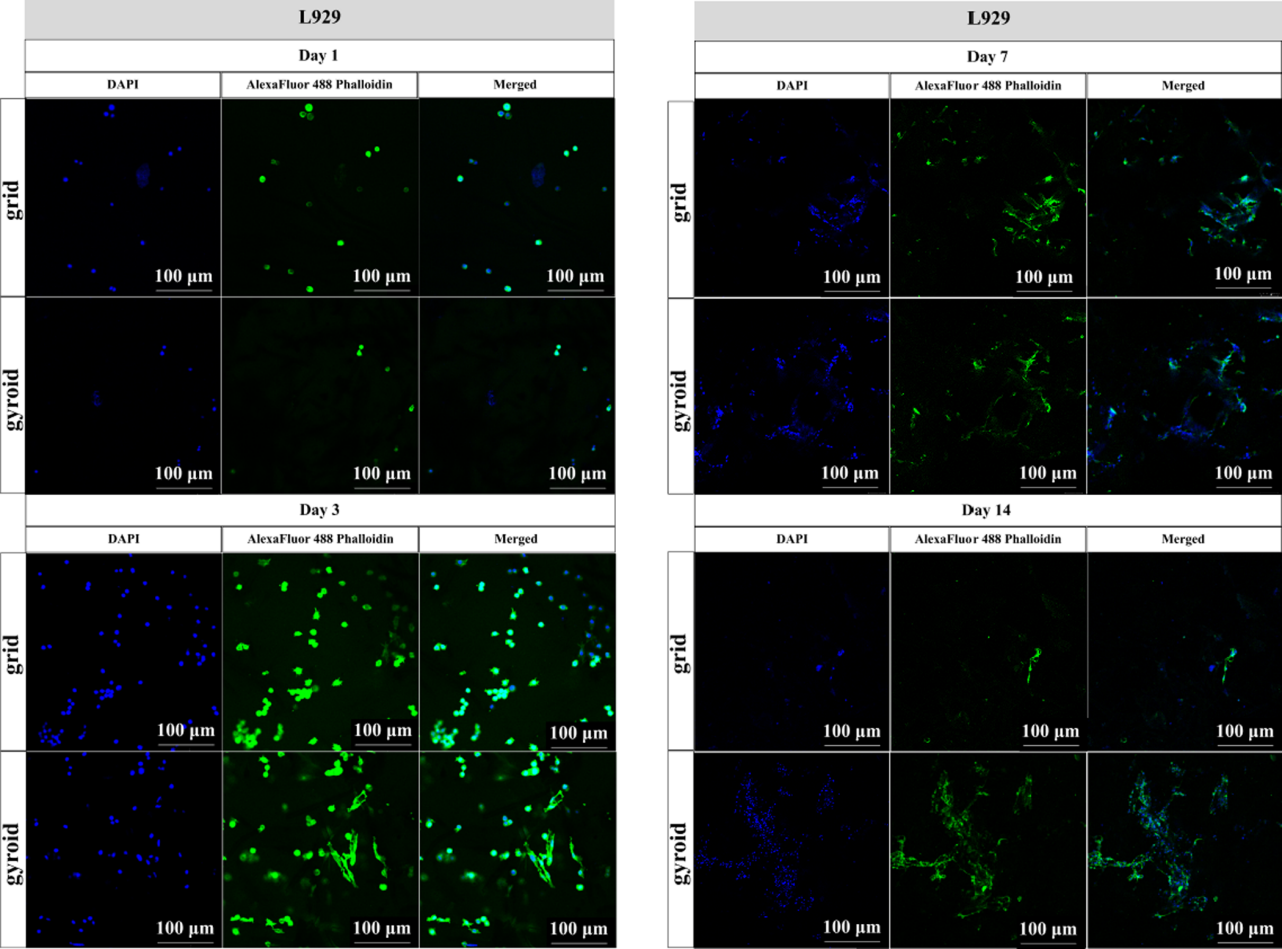
Confocal microscopy images of the grid and gyroid models (10× magnification). Gradient of L929 cell adhesion on the grid and gyroid model scaffolds was determined according to experimental times of 1, 3, 7, and 14 days.

Figure 10 shows the confocal images obtained from the cell adhesion assay on grid and gyroid scaffolds with the MC3T3-E1 cells at experimental times of 1, 3, 7, and 14 days. The image reveals that on day 1, the gyroid model exhibited a higher cell adhesion compared to the grid model. By day 3, this trend continued, with the gyroid model showing a more extensive cell distribution and spreading. On day 7, the cell number started to decrease and continued until day 14. At this time point, the grid model had distinguishably fewer cells than the gyroid model, indicating that the gyroid structure provided a more favorable environment for MC3T3-E1 cell adhesion and growth.

**Figure 10:**
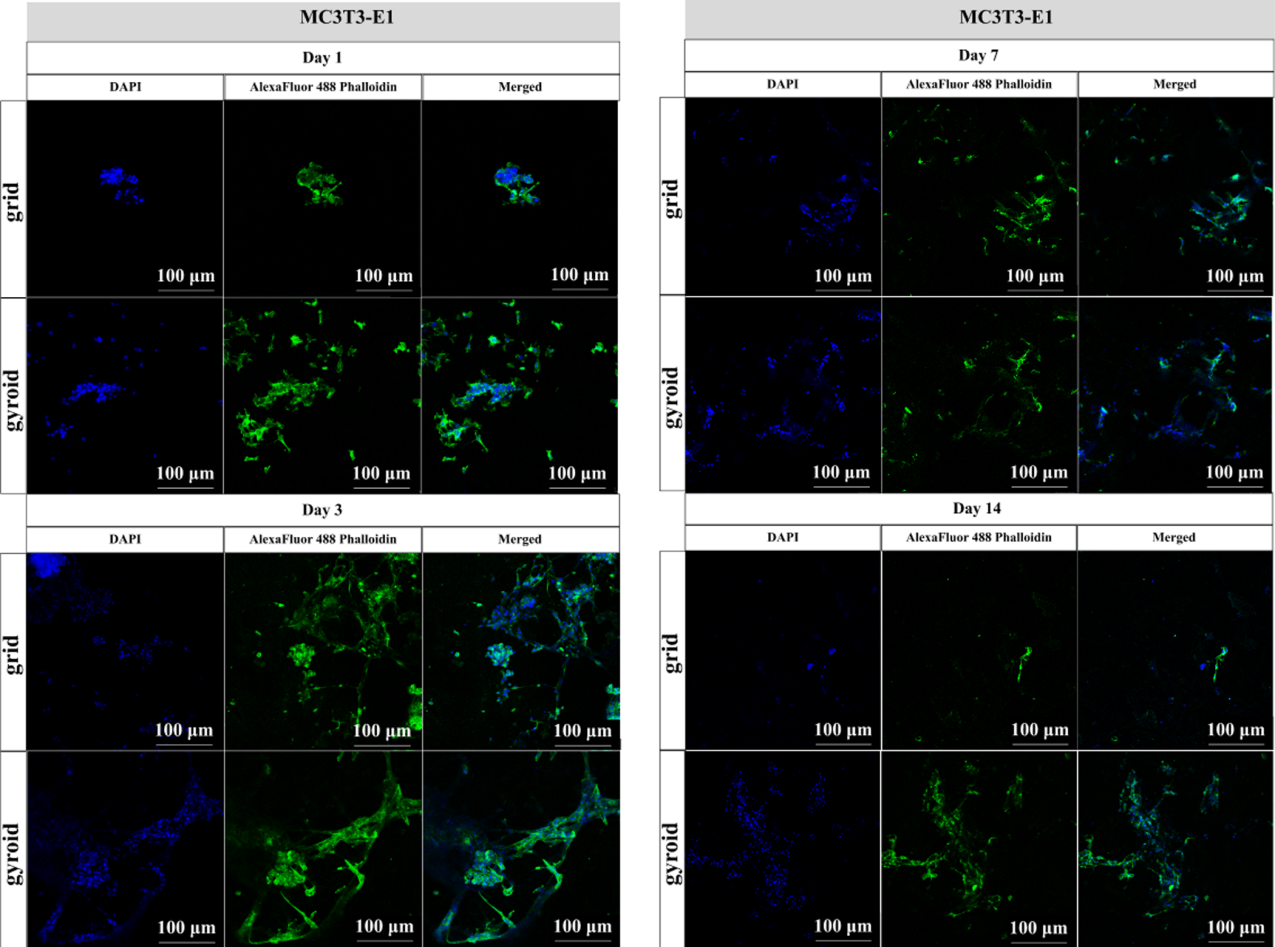
Confocal microscopy images of the grid and gyroid models (10× magnification). The gradient of MC3T3-E1 cell adhesion on the grid and gyroid model scaffolds was calculated according to experimental times of 1, 3, 7, and 14 days.

## Discussion

This study aimed to manufacture 3D printed scaffolds in two different models (grid and gyroid models), with BS extracted from the marine sponge Dragmacidon reticulatum, and to study their physicochemical and mechanical characteristics and the biological effects in in vitro tests. SEM analysis demonstrated the morphology of BS spicules and the interconnected pores for both scaffolds, with the grid model showing a porosity slightly higher than that of the gyroid model. From the results of the present work, it could be noted that a decrease in pH values was observed for both scaffolds up to 14 days postincubation, and the mass loss of the gyroid model was higher. FTIR and EDS demonstrated characteristic peaks of the alginate and BS (such as O–H, C=O, and C, and Si), and higher values in the compression test were observed for the gyroid model. Moreover, the in vitro studies demonstrated that both scaffolds were able to support cell integration for both scaffolds, but with a greater cell adhesion for the gyroid model.

The use of BS from marine sponges for manufacturing scaffolds for bone tissue engineering proposals has been considered a goldmine.^10^ Many authors state that BS presents biocompatibility, similarity with the natural extracellular matrix, tunable chemistry, and lower production costs compared to other synthetic materials.^10,32^ Moreover, in this study, 3D-printed BS scaffolds were manufactured and compared. In the SEM analysis, similar findings were seen for both models, with the clear presence of BS spicules presenting degradation after incubation. Similarly, Sousa et al.^34^ also observed the presence of BS spicules in a sharp format and a thin and homogeneous layer of ALG surrounding the BS particles in 3D printed BS/ALG scaffolds.

In the present study, both scaffolds presented porosity values higher than 80%, which may indicate scaffolds with a more suitable structure for supporting bone cell proliferation and tissue ingrowth.^35^ A porosity ranging from 50 to 90% (similar to trabecular bones) allows adequate diffusion of nutrients to cells and supports cell growth.^36–38^ Kido et al.^39^ obtained in their study a scaffold manufactured with Bioglass with a porosity of 80%, stating that this value was appropriate to provide enough space for cell ingrowth and transport of nutrients, oxygen, and growth factors. Sousa et al.^34^ observed a porosity ranging from 40 to 75% in BS 3D-printed scaffolds, highlighting the potential of the reference samples as an optimized candidate to be used as a bone graft.

Moreover, higher mechanical properties were observed for the gyroid model, showing higher resistance to maximum force loads, possibly due to its wavy geometry, which provided a higher interconnectivity between the stronger filaments, leading to a higher resistance compared to the grid model. It is known that the grid structure is the simplest structure used for bone scaffolds. It is constituted by layers, with uniform pore distribution and possibly, the stress concentrations at the intersection nodes of the model grid negatively influencing its mechanical performance.^17,36^ Conversely, for the gyroid model, the structural design, such as pore size, shape, and porosity, can be controlled by adjusting the parameter to simulate the porous structure of natural bone. Therefore, this model may be more suitable for constructing bone scaffolds. The findings of the present work corroborate those of Guo et al.,^38^ who demonstrated that in the compression test, the gyroid model showed higher compression strength than the grid structure, which was attributed to the continuous curved structure, which alleviated stress concentration and had a more uniform stress bearing. It is stated that the continuous rate of curvature of gyroids, removing the nodal points which may favor cracks initiation in the gyroid geometry, produces a superior fatigue resistance of this model.^40^ This fact can explain the higher mechanical properties of the gyroid scaffold.

A significant reduction in pH was obtained for both scaffolds after 14 days of immersion, with higher values found for the gyroid model. These results corroborate those found by Gabbai-Armelin et al.,^14^ who also observed a decrease in the values found for the pH of BS samples after 14 days of immersion. Sousa et al.^34^ also demonstrated that 3D printed BS scaffolds presented a significant decrease in pH and mass loss over time after incubation. In the present study, a linear decrease of pH was observed on day 1 for both samples, stabilizing at day 7, keeping the same values on day 14. A homeostatic pH is necessary for cell survival, and it is known that an excessive alkaline or acidic biological environment is highly harmful for cells, leading to cell death. It is well-known that once bioceramics and bioglasses are in contact with fluids, a release of ions occurs, especially silica, sodium, and calcium, resulting in an increased pH.^13^ It is suggested that this decrease in pH is related to the degradation of sodium alginate, which is composed of a carboxylate group that binds to other ions and molecules, forming hydrogen bonds.^14^ By combining ALG/BS, the acidic and basic degradation products apparently counteracted each other, resulting in a more homeostatic environment.

Furthermore, an intense mass degradation was observed mainly in the gyroid model, reaching around 70% of the initial mass on day 14, a behavior that was not observed in the grid model, suggesting that the different scaffolds present different mass stability. The rate of biomaterial degradation and mass loss is a very important variable for the success of the bone graft due to the need for liberation of space into the fracture site, allowing the ingrowth of newly formed bone tissue.^36^ Also, it is known that a rapid ion release is initiated immediately after the contact of BS with fluids, starting the degradation of the material, which could have contributed to the intense mass loss, especially in the gyroid model. Taken together, these data indicated that the behavior of degradation of the gyroid model may culminate in a biological advantage, with an accelerated dissolution of ions from the scaffold and a faster liberation of space, stimulating a higher formation of tissue ingrowth.

The FTIR analysis demonstrated both models presented similar compositions, with the characteristic peaks of BS, comprising Si–O–Si stretching, Si–O bending vibrations, and out-of-plane Si–O bending vibrations.^14,25^ Similarly, the characteristic peaks of sodium alginate were found, including O–H stretching vibrations, asymmetric and symmetric C=O stretching vibrations, and C–O–H and C–O–C functional groups.^22–24^ The relative amounts of elements in the EDS analysis, which was carried out on scaffolds submerged in SBF solution, differed between the groups. On day 0, the gyroid model had the elements C, O, and Si. In contrast, the lattice model included Cl and Ca, both of which may be the result of cross-linking. Thus, it is suggested that the grid model may have more residual material during washing after cross-linking compared to the gyroid model. On subsequent days, Si was more present in the grid model than in the gyroid model. It is worth noting that the elements may interact with the SBF solution and influence their deposition on the scaffolds as well as intensify their dilution over the experimental periods. It can also indicate that even though extraction is carried out with steps to ensure that all elements other than silicon are degraded and removed, there may still be residues that can be observed in the EDS analysis.

The in vitro cell adhesion assay demonstrated that the gyroid-shaped scaffold model presented an increased number of fibroblast and osteoblast cells compared to the grid model along the experimental periods, indicating that the gyroid structure provided a more favorable environment for cell ingrowth. Guo et al.^38^ found, through in vitro experiments, a higher number of cells on the gyroid scaffold when compared to the grid porous scaffold model, with better cell adhesion and proliferation also in the gyroid scaffold. Also, Sousa et al.^34^ demonstrated through in vitro studies that 3D printed BS scaffolds had positive effects on osteoblast cell proliferation and nongenotoxic effects. These authors stated that BS was a compatible material, able to produce an increase in cell viability.^41^ It seems that the composition and the structure of both scaffolds, especially the gyroid model, showed a proper structure to support cell ingrowth and proliferation, possibly constituting a bone graft with improved biological properties.^42–44^

The optimization of the structure and morphology of 3D-printed scaffolds for bone tissue engineering is in high demand. In the present study, grid and gyroid models made with marine BS had their morphologies and in vitro effects compared, demonstrating a clear indication of the superiority of the gyroid model. However, further studies involving more detailed in vitro experiments and preclinical studies remain to be performed to continue the investigation of the gyroid-shaped scaffolds manufactured with BS.

## Conclusions

In conclusion, 3D printing proved to be effective in fabricating both BS scaffolds, with the gyroid model showing superior mechanical strength and increased adhesion of fibroblasts and osteoblasts throughout the study. These findings highlight the potential of gyroid scaffolds for bone tissue engineering proposals. Further in vivo studies need to be performed in order to investigate these promising properties of the scaffolds in critical bone defects for supporting their application in tissue engineering and confirming their regenerative capabilities.

## Abbreviations

BS: biosilica
SEM: Scanning electron microscopy
FTIR: Fourier transform infrared spectroscopy
EDS: Energy dispersive X-ray spectroscopy
ALG: alginate
Si: silicon
Cl: chlorine
Ca: calcium
NaClO: sodium hypochlorite
HNO_3_: nitric acid
H_2_SO_4_: sulfuric acid
CaCl_2_: calcium chloride
MEM-α: Minimum essential medium alpha
FBS: Fetal Bovine Serum
DMEM: Dulbecco’s modified Eagle’s medium
PFA: Perfluoroalkoxyalkanes
PBS: phosphate-buffered saline
mL: milliliter
μm: micrometer
RPM: revolutions per minute
w/v: weight/volume
mm: millimeter
mm/s: milimter/second
h: hour
nm: nanometer
SE: secondary electron
kV: kilovolt
mm/min: milimer/min
mM: millimolar
cm: centimeter
CO_2_: carbon dioxide
DNA: nuclear DNA
N: newton
kPa: kilopascal
*F*_max_: maximum strength
σ_max_: maximum voltage
C: carbon
O: oxygen
SBF: simulated body fluid
